# A Study to Improve the Performance of Mixed Cation–Halide Perovskite-Based UVC Photodetectors

**DOI:** 10.3390/nano12071132

**Published:** 2022-03-29

**Authors:** Ga In Choi, Hyung Wook Choi

**Affiliations:** Department of Electrical Engineering, Gachon University, 1342 Seongnam-daero, Seongnam-si 13120, Korea; gana034@gachon.ac.kr

**Keywords:** mixed cation, mixed halide, perovskite device, UVC sensor, photodetector

## Abstract

Photodetectors convert optical signals into electrical signals and demonstrate application potential in various fields, such as optical communication, image detection, environmental monitoring, and optoelectronics. In this study, a mixed cation–halide perovskite-based ultraviolet C photodetector was fabricated using a solution process. The higher the mobility of the perovskite carrier, which is one of the factors affecting the performance of electronic power devices, the better the carrier diffusion. The on/off ratio and responsivity indicate the sensitivity of the response, and together with the detectivity and external quantum efficiency, these parameters demonstrate the performance of the detector. The detector fabricated in this study exhibited a mobility of 202.2 cm^2^/Vs and a high on/off ratio of 10^5^% at a −2 V bias, under 254 nm light irradiation with an intensity of 0.6 mW/cm^2^. The responsivity, detectivity, and external quantum efficiency of the as-fabricated detector were 5.07 mA/W, 5.49 × 10^11^ Jones, and 24.8%, respectively. These findings demonstrate that the solution process employed in this study is suitable for the fabrication of mixed cation–halide perovskites which show immense potential for use as photodetectors.

## 1. Introduction

A photodetector is a device that converts an optical signal into an electrical signal and exhibits a high detection rate, allowing the detection of weak optical signals [1,2]. Photodetectors have been used in various fields, such as optical communication, image detection, and environmental monitoring [3,4,5,6]. In particular, ultraviolet C (UVC) photodetectors operating in the 280–100 nm region have been intensively studied owing to their ability to precisely detect weak signals [7]. UV rays can be divided into three categories according to wavelength: UVA (400–320 nm), UVB (320–280 nm), and UVC (280–100 nm) [8]. UVC radiation is largely absorbed by the ozone layer [8]. However, UVC radiation is emitted by artificial light sources, such as mercury lamps, UV germicidal bulbs, and arc discharges [9,10,11,12], and is harmful to human health. Additionally, it is the cause of many safety issues in industrial facilities [13,14]. Therefore, it is essential to develop a system to detect UVC radiation in order to increase worker safety and reduce facility management costs.

When fabricating a UVC photodetector, it is necessary to consider whether a low-temperature and simple process is possible. This is because it is essential to develop sustainable electronic devices to reduce the impacts on the environment of energy consumption and waste [15,16]. Low energy processes make it possible to produce more eco-friendly devices [17].

Various characteristics of photodetectors, such as stability, light absorption, carrier mobility, band gap, reactivity, detectability, and on/off ratio, are dependent on the material composition. Accordingly, photodetectors comprising various materials have been investigated [1]. Photodetectors comprising materials such as group III–V compound semiconductors, organic polymers, and silicon have been fabricated [2], and narrow bandgap Si diode sensors equipped with UV filters have been developed [14]. However, photodetectors containing these materials are expensive and require complex fabrication processes [18]. Additionally, Si diodes offer limited UVC sensitivity because the photogenerated carriers cannot easily reach the depletion layer of the semiconductor [14].

Recently, perovskites have attracted attention in the field of optoelectronic devices, such as light-emitting diodes and solar cells [19,20], owing to their tunable bandgaps, large light absorption, and long diffusion lengths (*L_D_* values) [21]. In addition, the solution process fabrication of perovskites is simple and economical [22,23].

Methylammonium (MA) and formamidinium (FA) cations are commonly used in perovskite light absorption layers [21,24], and methylammonium lead halide (MAPbX_3_) has attracted attention as a material for developing hybrid organic–inorganic perovskites owing to its excellent photoelectric properties [25]. However, MAPbX_3_ perovskites exhibit phase instability at high temperatures and are more sensitive to moisture and light than FA-based perovskites, leading to the separation of halide components [26,27]. Formamidinium lead halides (FAPbX_3_) have a higher thermal stability than MAPbX_3_ [24,28]; however, FAPbI_3_ exhibits low phase stability at room temperature and can be converted from the “black” α-phase to the “yellow” δ-phase [29,30].

Therefore, perovskite materials comprising only pure cation MA or FA are not optimal owing to their instability. However, the incorporation of MA^+^ into FAPbI_3_-based perovskite via the crystal nucleation mechanism prevents the formation of δ-FAPbI_3_ [31], and this stabilization improves performance [32]. Additionally, the iodide can be partially substituted with a bromide to improve the performance of FA/MA mixed cation perovskites, as the addition of a small amount of Br^−^ improves phase stability [33,34]. The partial substitution of I^−^ with Br^−^ maintains excellent light absorption properties and increases the carrier lifetime and mobility [25]. The mixed cation–halide perovskite features improvements in crystallinity, stability, and electrical properties, such as light absorption, photocurrent, carrier mobility, and *L_D_*. Therefore, mixed cation–halide perovskites have potential as materials for use in high performance UVC photodetectors with a low energy process and cost.

In this study, (FAPbI_3_)_1−x_(MAPbBr_3_)_x_ (x = 0, 0.03, 0.05, 0.07, and 0.1) photodetectors were fabricated by adding MAPbBr_3_ to FAPbI_3_, considering the low thermal stability of MABr at different mol% ratios. The fabricated mixed cation–halide perovskite-based photodetector exhibited improved stability, photocurrent, and responsiveness compared with a pure FAPbI_3_ photodetector. The performance of the photodetector was determined by investigating various characteristics, including responsivity, detectivity, carrier mobility, light absorption, dark environment, and current under 254 nm irradiation. The responsivity, detectivity, and external quantum efficiency (*EQE*) of the as-fabricated (FAPbI_3_)_1−x_(MAPbBr_3_)_x_-based photodetectors were 5.07 mA/W, 5.49 × 10^11^ Jones, and 24.8%, respectively, under 254 nm light irradiation with a −2 V bias and 0.6 mW/cm^2^ intensity. This value is more than six times larger than that of a pure FAPbI_3_ photodetector. These results show that the mixed cation–halide perovskite provides improved optical properties compared with the pure FAPbI_3_ perovskite, and therefore, is a promising candidate for UVC photodetector fabrication.

## 2. Materials and Methods

### 2.1. Materials

The materials and chemicals used were as follows: quartz indium tin oxide (ITO) patterned glass (etching interval: 10 μm) with a thickness of 150 nm, lead (II) iodide (99.999% trace metal basis), *N*,*N*-dimethylformamide (DMF, 99.8%), toluene (99.8%), dimethyl sulfoxide (DMSO, 99.7%), FA iodide (FAI), MA iodide (MAI), and MA chloride (MACl).

### 2.2. Preparation of (FAPbI_3_)_1−x_(MAPbBr_3_)_x_-Based Perovskite Photodetector

**Figure 1:**
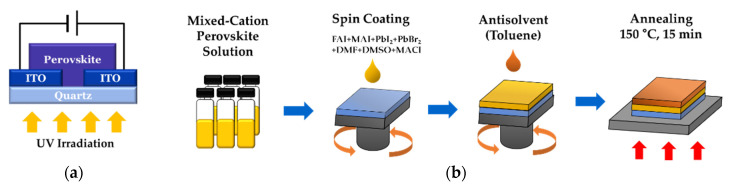
(**a**) Schematic and (**b**) fabrication process of the (FAPbI_3_)_1−x_(MAPbBr_3_)_x_ photodetector.

Figure 1a shows a schematic diagram of a metal-semiconductor-metal-type photodetector, and Figure 1b illustrates the fabrication process. An ITO quartz substrate etched at 10 µm intervals was used to prepare the (FAPbI_3_)_1−x_(MAPbBr_3_)_x_ films. To remove organic matter, the ITO quartz substrate was ultrasonically cleaned using a neutral detergent, water, acetone, ethanol, and distilled water for 20 min each. After drying in an oven at 80 °C for 20 min, UV-ozone treatment was performed for 30 min to further remove moisture. A (FAPbI_3_)_1−x_(MAPbBr_3_)_x_ solution was prepared by dissolving PbI_2_ and PbBr_2_ (1.4 M), FAI, and MABr (1.4 M) in DMF (1 mL), which was then mixed with 100 µL of DMSO. The perovskite precursor solution was then stirred for 1 h at 23 °C. MACl was then added, up to 40 mol% of FAI, and the solution was further stirred for 30 min. The solution was deposited on a quartz substrate via spin coating at 4000 rpm for 20 s, over which toluene was dropped for the last 10 s. After spin-coating, the thin film was annealed at 150 °C for 15 min.

### 2.3. Device Characterization

The crystal structure of the perovskite film was investigated using X-ray diffraction (XRD, DMAX 2200, Rigaku, Japan) with a scan rate of 3.00°/min. The morphology of the photodetector surface was observed using scanning electron microscopy (SEM, S-4700, Hitachi, Japan). The light absorptivity of the device was measured using UV-visible (UV-vis) spectroscopy (UV-vis 8453, Agilent, Santa Clara, CA, USA). The electrical response of the perovskite photodetector was measured with a combined source and measurement meter (Source Measure Unit, Keithley Instruments, Cleveland, OH, USA) and a UV lamp (6 W, 254 nm) (VL-6.LC, Vilber Lourmat, Marne-la-Vallée, France) under 254 nm irradiation.

## 3. Results and Discussion

**Figure 2:**
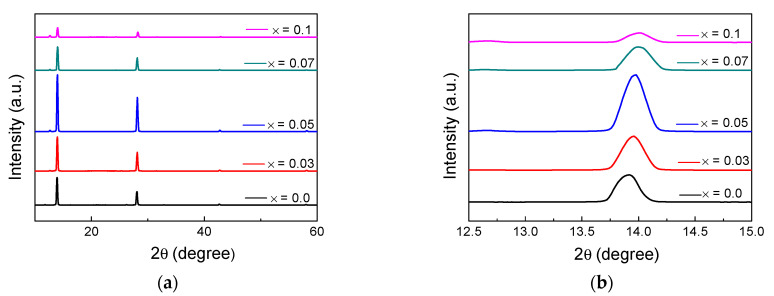
Crystal structure of (FAPbI_3_)_1−x_(MAPbBr_3_)_x_ films. (**a**) XRD patterns for different composition ratios (x = 0, 0.03, 0.05, 0.07, and 0.01) and (**b**) XRD patterns in the 13.0–15.0° range.

Figure 2a shows the XRD patterns of the fabricated perovskites. (FAPbI_3_)_1−x_(MAPbBr_3_)_x_ films were fabricated with different ratios of x (x = 0, 0.03, 0.05, 0.07, and 0.1). Several characteristic perovskite peaks were observed in the XRD patterns. The peaks at ~11.7°, ~12.7°, and ~14.0° of the diffractogram were assigned to the δ-FAPbI_3_, PbI_2_, and α-FAPbI_3_ phases, respectively. Pure FAPbI_3_ (x = 0) exhibited the highest δ-FAPbI_3_ peak intensity and possessed low phase stability owing to the high concentration of δ-FAPbI_3_. As the MAPbBr_3_ content of the film increased, δ-FAPbI_3_ was converted to α-FAPbI_3_ [25], which decreased the peak intensity of δ-FAPbI_3_. However, the reduction of the δ-FAPbI_3_ content did not improve the crystallinity of the all perovskite films. Although the α-FAPbI_3_ peak intensity of the (FAPbI_3_)_1−x_(MAPbBr_3_)_x_ film increased up to x = 0.05, it started to decrease at x = 0.07 and exhibited the smallest peak intensity at x = 0.1. This is because the low thermal stability of MABr led to the degradation of MAPbBr_3_. As the “black” α-FAPbI_3_ phase is thermodynamically stable above 150 °C, FAPbI_3_ was annealed at 150 °C [32]. However, MA^+^ has low thermal stability [26], and therefore, at high temperatures, a portion of MAPbBr_3_ thermally decomposes to form a PbI_2_ phase [32,35]. The highest PbI_2_ peak intensity was observed when x = 0.1, as shown in Figure 2a.

Figure 2b shows that as the content of (MAPbBr_3_)_x_ increased, the peak shifted to a high diffraction angle. This shows that the perovskite films were successfully formed, as lattice shrinkage is expected to occur when FA^+^ (2.79 Å) is replaced with MA^+^ (2.70 Å) [35]. Moreover, additional lattice shrinkage is expected when Br^−^ (1.96 Å) replaces I^−^ (2.2 Å), which shifts the corresponding peak to high diffraction angles [32,36].

The results shown in Figure 2 demonstrate that when mixed cation–halide perovskites were prepared, the content of δ-FAPbI_3_ decreased compared to that of the pure FAPbI_3_, and the phase stability improved. In particular, the crystallinity of (FAPbI_3_)_0.95_(MAPbBr_3_)_0.05_ was the highest among the synthesized samples. However, when more than 7 mol% MAPbBr_3_ was added, a decrease in crystallinity, owing to the formation of a residue, was observed. This indicates that the overall crystallinity varies depending on the composition of the mixed cation perovskite.

**Figure 3:**
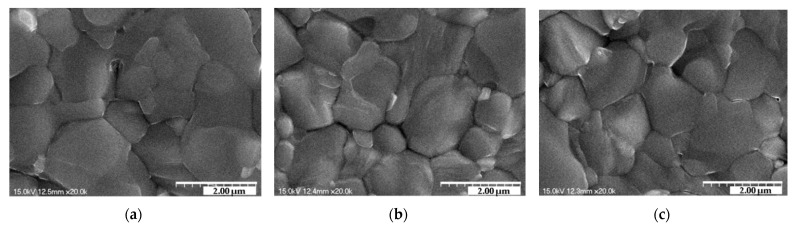

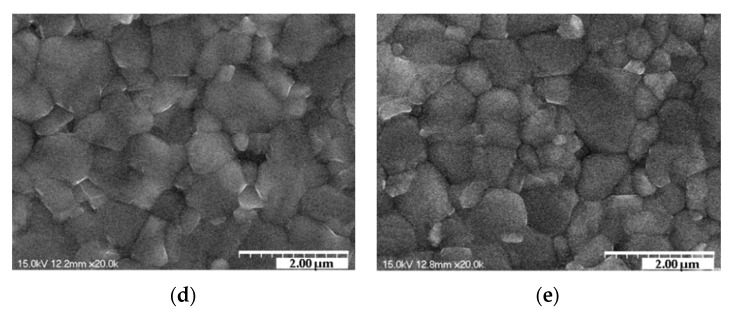
SEM images of (FAPbI_3_)_1−x_(MAPbBr_3_)_x_ films for different composition ratios: (**a**) x = 0, (**b**) x = 0.03, (**c**) x = 0.05, (**d**) x = 0.07, and (**e**) x = 0.1.

Figure 3 displays SEM images of the (FAPbI_3_)_1−x_(MAPbBr_3_)_x_ perovskite films; it confirms that a change in the composition ratio x affects the film surface. The average diameter lengths of the grain of (FAPbI_3_)_1−x_(MAPbBr_3_)_x_ films (x = 0, 0.03, 0.05, 0.07, 0.1) were 1.629, 1.496, 1.465, 0.922, and 0.815 um. In Figure 3a–c, the grain size decreased as x increased. This is because MA^+^ (2.70 Å) has a smaller particle size than FA^+^ (2.79 Å), and Br^−^ (1.96 Å) has a smaller particle size than I^−^ (2.2 Å) [32,35]; as such, when x increases, more MA^+^ and Br^−^ replace FA^+^ and I^−^ [36]. However, compared to when x = 0.07, the difference in the small grain diameter increase was not significant.

Figure 3d,e show the sharply reduced grain size. Additionally, a residue was observed when x = 0.1 because a portion of MAPbBr_3_ was decomposed, at because of the high annealing temperature of 150 °C, forming PbI_2_ and PbBr_2_. This suggests that the perovskite was not well-formed at this composition ratio. A small grain size with many crystals observed on the surface leads to deterioration of performance. As residues are produced and replaced with smaller ions, resulting in a smaller grain size, the mobility of carrier transport decreases, which, in turn, increases carrier scattering, leading to poor performance [37].

**Figure 4:**
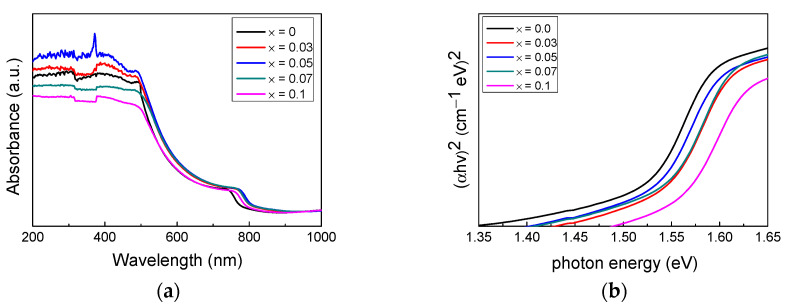
Light absorption and bandgap of (FAPbI_3_)_1−x_(MAPbBr_3_)_x_ films (x = 0, 0.03, 0.05, 0.07, and 0.01). (**a**) UV-vis absorbance spectra and (**b**) Tauc plot.

Figure 4a presents the optical absorption spectra of the (FAPbI_3_)_1−x_(MAPbBr_3_)_x_ films (x = 0, 0.03, 0.05, 0.07, and 0.1). All (FAPbI_3_)_1−x_(MAPbBr_3_)_x_ samples displayed a strong absorbance at 200–500 nm. This demonstrates that the (FAPbI_3_)_1−x_(MAPbBr_3_)_x_-based photodetector sufficiently detected light in the UVC region. The absorbance increased as the value of x increased but decreased when the value of x exceeded 0.07. This suggests that certain (FAPbI_3_)_1−x_(MAPbBr_3_)_x_ compositions (x = 0.03 and 0.05) have better light absorption characteristics than pure FAPbI_3_.

Figure 4b is an estimated Tauc plot based on the light absorption of the (FAPbI_3_)_1−x_(MAPbBr_3_)_x_ (x = 0, 0.03, 0.05, 0.07, and 0.1) films. The sharp band energies indicate the presence of a direct bandgap. Semiconductors with wide bandgaps can absorb and emit high-energy photons, enabling the fabrication of high-performance photonic devices, including UV detectors [38]. Based on the light absorption characteristics, the bandgap of pure FAPbI_3_ and (FAPbI_3_)_1−x_(MAPbBr_3_)_x_ (x = 0.03, 0.05, and 0.07) was estimated to be 1.45 eV and 1.52 eV, respectively. The increase in the band gap was due to the structural distortion caused by the stress on the Pb–I bond resulting from the incorporation of Br [36]. Changes in the kinetic energy of the electron-hole pairs due to Coulomb interactions also result in bandgap differences [39]. The largest bandgap of 1.55 eV was observed when x = 0.1. However, PbI_2_ residues are likely to be present when x = 0.1 [36].

**Figure 5:**
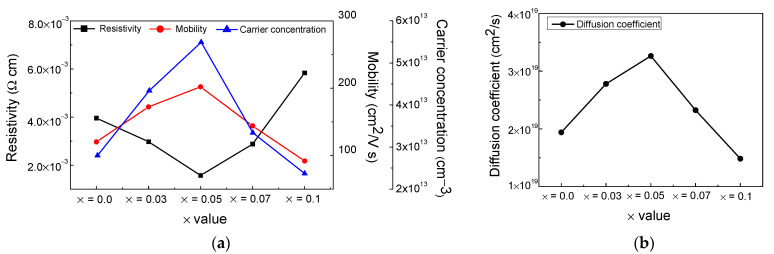
(**a**) Comparison of electrical characteristics including resistivity, carrier concentration and mobility, (**b**) diffusion coefficients of (FAPbI_3_)_1−x_(MAPbBr_3_)_x_ films (x = 0, 0.03, 0.05, 0.07, and 0.01).

Figure 5a shows the electrical properties (resistivity, mobility, and carrier concentration) of the (FAPbI_3_)_1−x_(MAPbBr_3_)_x_ thin films (x = 0, 0.03, 0.05, 0.07, and 0.1). The resistivity of (FAPbI_3_)_1−x_(MAPbBr_3_)_x_ decreased as the x value increased, except for x = 0.1. In particular, (FAPbI_3_)_0.95_(MAPbBr_3_)_0.05_ displayed the lowest resistivity, i.e., 0.00157 Ω cm.

Carrier concentration and mobility are significant parameters for semiconductor materials and electronic power devices [40]. All (FAPbI_3_)_1−x_(MAPbBr_3_)_x_ films had carrier concentration values higher than 10^13^ cm^−3^. The current depends on the mobility; the higher the mobility, the higher the current [38,41,42]. The mobilities of the (FAPbI_3_)_1−x_(MAPbBr_3_)_x_ films were 120.3, 172.2, 202.2, 144, and 91.9 cm^2^/V.s for x values of 0, 0.03, 0.05, 0.07, and 0.1, respectively.

Figure 5b shows the diffusion coefficient (*D*) of the (FAPbI_3_)_1−x_(MAPbBr_3_)_x_ films (x = 0, 0.03, 0.05, 0.07, and 0.1). *D* is related to the mobility, as shown in Figure 5a. Diffusion involves the movement of charge carriers driven by a concentration gradient [14,43]. In the presence of a magnetic field, *D* is defined using the Einstein relation *D* = μK*T*/*q*, where μ is mobility, K is the Boltzmann constant, *T* is the temperature of the sample, and *q* is the amount of charge [43]. The *D* of the (FAPbI_3_)_1−x_(MAPbBr_3_)_x_ films increased as the x value increased, except for x = 0.1. The charge carrier *L_D_* was calculated as *L_D_* = √*Dτ*, where *τ* is the lifetime [44]. The mobility and *L_D_* are proportional, and high mobility leads to an increase in the *D* and *L_D_* values. When I^−^ is replaced by Br^−^, the lattice parameters shrink, leading to an increase in the photogenerated carrier lifetime and the charge carrier mobility [33,45]. Combining the results in Figure 5, it shows that the (FAPbI_3_)_1−x_(MAPbBr_3_)_x_ film had the best electrical properties such as carrier concentration and mobility when x = 0.05.

In addition, to analyze the performance of the (FAPbI_3_)_1−x_(MAPbBr_3_)_x_ (x = 0, 0.03, 0.05, 0.07, and 0.1) photodetectors, the current-voltage (*I*–*V*) characteristics were measured from −2 V to 2 V at a scan rate of 0.1 V. Measurements were performed in a dark environment and under a 254 nm light source with an output of 0.6 mW/cm^2^. In the perovskite photodetector, a Schottky barrier formed owing to the contact with the ITO electrode, and the current flowed through it.

**Figure 6:**
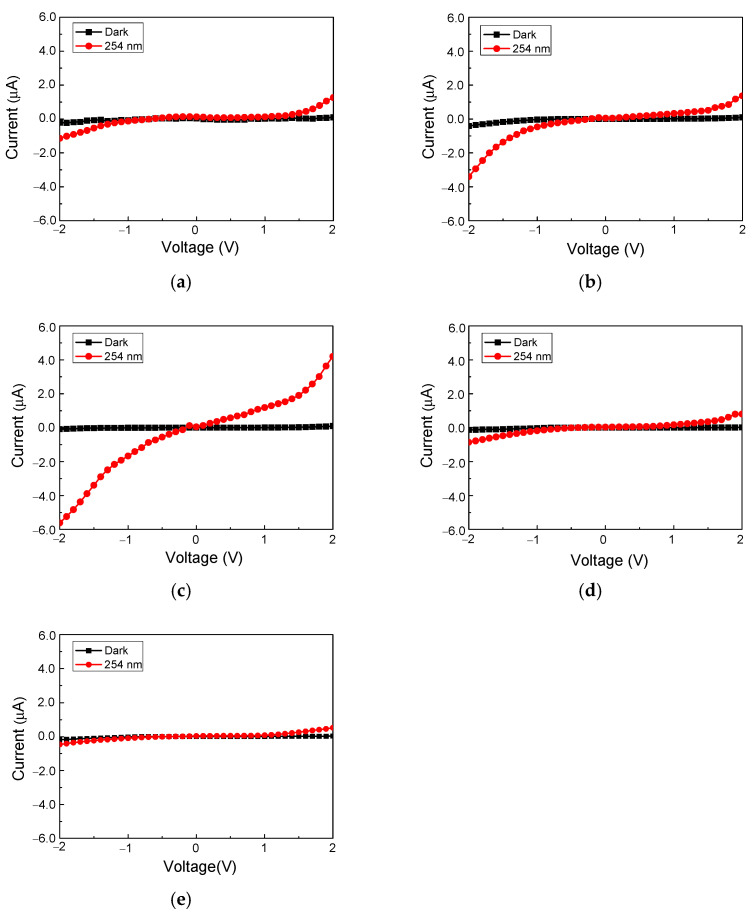
Current–voltage characteristics of (FAPbI_3_)_1−x_(MAPbBr_3_)_x_ photodetectors for different composition ratios: (**a**) x = 0, (**b**) x = 0.03, (**c**) x = 0.05, (**d**) x = 0.07, and (**e**) x = 0.1.

Figure 6a–e show that the(FAPbI_3_)_1−x_(MAPbBr_3_)_x_ photodetectors were barely able to detect 254 nm light at 0.6 mW/cm^2^ in a dark environment. However, the (FAPbI_3_)_0.95_(MAPbBr_3_)_0.05_ photodetector generated significant photocurrents of −5.61 µA at −2 V and 4.2 µA at 2 V under 254 nm irradiation with an output of 0.6 mW/cm^2^, as shown in Figure 6c.

**Figure 7:**
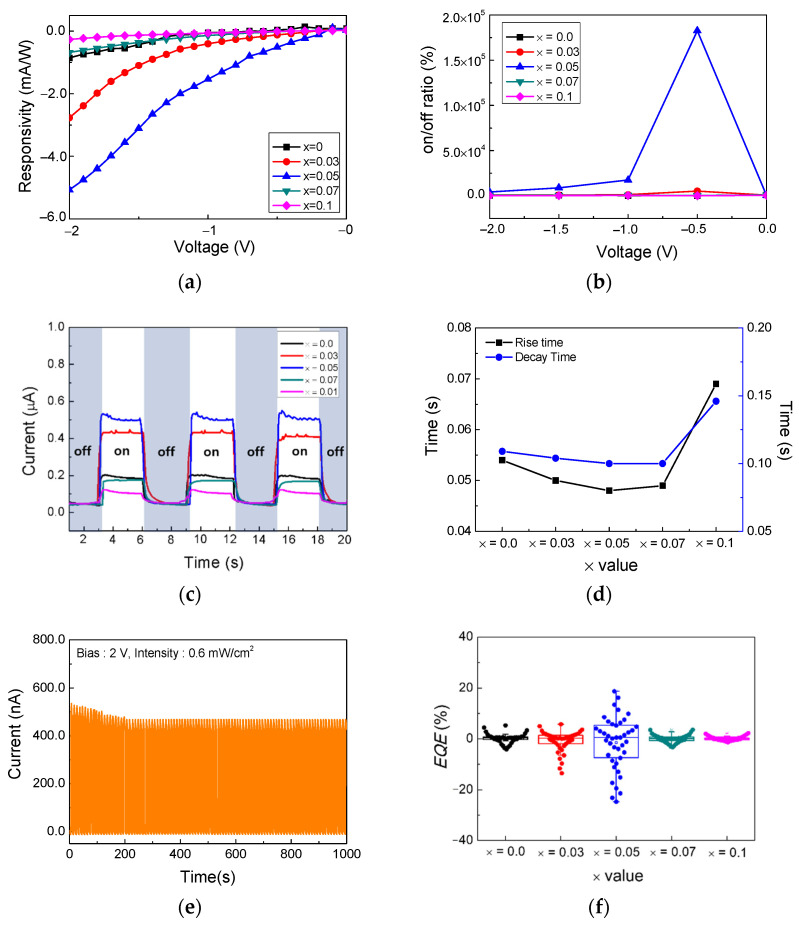
(FAPbI_3_)_1−x_(MAPbBr_3_)_x_ photodetector response. (**a**) Responsivity curves, (**b**) on/off ratio, (**c**) transient photo-responses, (**d**) rise and decay times, (**e**) photostability of the prepared device switching under the illumination of 254 nm light with an intensity of 0.6 mW/cm^2^, and (**f**) external quantum efficiency.

To evaluate the performance of the photodetector, the significant parameters of the detector under a light of 254 nm with an output of 0.6 mW/cm^2^ were investigated. Figure 7a shows the responsivity (*R*) of the films. *R* is the main parameter that indicates the performance of the photodetector and the response of the device to the irradiated light. *R* is defined as *R* = (*I_254_* − *I_dark_*)/*P* × *S* mA/W [14], where *I_254_* is the current when irradiated with 254 nm light, *I_dark_* is the dark environment current, *P* is the power of the incident light, and *S* is the irradiated area of the device [46]. *R* gradually decreased as the bias decreased owing to the change in the photon-charge conversion efficiency of the photodetector [10]. Under 254 nm illumination, with an output of 0.6 mW/cm^2^, the *R* values of (FAPbI_3_)_1−x_(MAPbI_3_)_x_ were 0.85, 2.77, 5.07, 0.68, and 0.26 mA/W at −2 V when x was 0, 0.03, 0.05, 0.07, and 0.1, respectively.

Figure 7b shows the on/off ratios of the (FAPbI_3_)_1−x_(MAPbBr_3_)_x_ photodetectors in the reverse bias region from −2 V to 0 V. A high on/off ratio is related to good performance in a photodetector [11]. The on/off ratios at −2 V were 539, 835, 4211, 663, and 258% for x = 0, 0.03, 0.05, 0.07, and 0.1, respectively. Additionally, when the voltage was −0.5 V, the largest on/off ratio was observed for x = 0.05.

Figure 7c,d show the time dependent response of the (FAPbI_3_)_1−x_(MAPbBr_3_)_x_ photodetectors. An on/off switching test was performed to observe transient photoresponses under 254 nm radiation with an 0.6 mA/cm^2^ intensity at a 2 V bias. Response times, such as rise and decay times, could be inferred from the relative speed of response of the detector to changes in the optical signal. Figure 7c shows that during on/off switching, the (FAPbI_3_)_1−x_(MAPbBr_3_)_x_ (x = 0.03 and 0.05) photodetectors exhibited a higher photo-response than pure FAPbI_3_. In addition, Figure 7d shows that when the x value was 0.05, the (FAPbI_3_)_1−x_(MAPbBr_3_)_x_ photodetectors had the smallest rise and decay times, i.e., 0.048 and 0.1 s, respectively. Therefore, the (FAPbI_3_)_0.95_(MAPbBr_3_)_0.05_ photodetector had the fastest photo-response among all the photodetectors, and is highly sensitive.

Figure 7e shows the on/off repeatability of the (FAPbI_3_)_0.95_(MAPbBr_3_)_0.05_ photodetector. The on/off switch was irradiated for 1000 s using a 254 nm light source with an output of 0.6 mW/cm^2^. In the first on/off iteration, the photocurrent was 523 nA. The light was irradiated for 5 s, and the photodetector was cycled on/off 100 times. After 100 cycles, the photocurrent decreased slightly to 468 nA, but remained constant from the 16th cycle to the 100th cycle. Although a slight decrease in photocurrent was observed, the maintenance of the photocurrent over a relatively long time proves its reproducibility.

*EQE*, which is the number of electrons produced per incident photon, is another important parameter that indicates the detector performance. *EQE* is defined as *Rh*c/e*λ*, where *R* is the responsivity, *h* is the Planck constant, c is the speed of light, e is the electron charge, and *λ* is the wavelength of the light [9]. Figure 7f shows the *EQE* from −2 V to 2 V bias as a box plot under 254 nm light irradiation with 0.6 mA/cm^2^ intensity. Figure 7c shows that the *EQE* increases up to x = 0.05 and decreases at x = 0.07. These results exhibit the same pattern as the other measurements (XRD, UV-vis, and responsivity measurements) and demonstrate that among the (FAPbI_3_)_1−x_(MAPbBr_3_)_x_ (x = 0, 0.03, 0.05, 0.07, and 0.1) photodetectors, the (FAPbI_3_)_0.95_(MAPbBr_3_)_0.05_ composition exhibited the best optical properties.

Detectivity D* is an important photodetector parameter, defined as D* = *R*/(2*qJ_dark_*)^1/2^, where *R* is the responsivity, *q* is the electron charge, and *J_dark_* is the current density of the dark current [14,38]. In Table 1, parameters, such as *R*, *D*, and *EQE,* of UVC photodetectors fabricated using various materials are listed. The *R* and *EQE* values of the (FAPbI_3_)_0.95_(MAPbBr_3_)_0.05_ photodetector fabricated in this study were superior to those of photodetectors reported in other studies. This suggests that the perovskite-based UVC photodetectors fabricated in this study are promising candidates in the field of UVC sensing, as the perovskites can be fabricated using a simple process at lower temperatures than those of previously reported photodetectors. In addition, the *R* and *EQE* values were superior to those of perovskite-based photodetectors reported in other studies, while the D* values were similar. These results indicate that (FAPbI_3_)_0.95_(MAPbBr_3_)_0.05_ is a promising material for developing perovskite-based photodetectors.

## 4. Conclusions

Mixed cation–halide perovskite (FAPbI_3_)_1−x_(MAPbBr_3_)_x_ (x = 0, 0.03, 0.05, 0.07, and 0.1)-based photodetectors were investigated. (FAPbI_3_)_1−x_(MAPbBr_3_)_x_ films exhibited the most improved stability and light absorption when the MAPbBr_3_ ratio was 5 mol%. The (FAPbI_3_)_0.95_(MAPbBr_3_)_0.05_ UVC photodetector exhibited an *EQE* of 24.8%, a mobility of 202.2 cm^2^/Vs, a response of 5.07 mA/W, and a detection rate of 5.49 × 10^11^ Jones. These are excellent parameter values compared with previously reported values for perovskite-based photodetectors fabricated using the solution process. By applying the process described in this study, it is possible to fabricate a photodetector with improved optical properties through a low-energy process. This suggests that mixed cation–halide perovskite-based photodetectors have significant potential for use in UVC photodetectors.

## Figures and Tables

**Table 1:** Comparison of UVC various photodetector parameters.

Materials	Light (nm)	Bias (V)	*R* (mA/W)	D* (Jones)	*EQE* (%)	Ref.
(FAPbI_3_)_0.95_(MAPbI_3_)_0.05_	254	−2	5.07	5.49 × 10^11^	24.8	[In this study]
(FAPbI_3_)_1−x_(MAPbBr_3_)_x_	254	0	4.92	7.57 × 10^10^	-	[11]
MAPbBr_3_	254	2	4.57	1.02 × 10^13^	22.2	[14]
CsPbBr_3/_	254	2	0.24	1.1 × 10^9^	0.05	[47]
CaSnO_3_	255	0	2.25	1.56 × 10^10^	1.06	[48]
Ag/GQD/Au	254	5	2.1	9.59 × 10^11^	10.3	[49]
Mn_0.52_Zn_0.48_O	250	10	0.1	-	-	[18]
Nb:SrTiO_3_/Ga_2_O_3_	254	0	2.6	-	1.3	[7]
Au/ZnS QDs/Au	254	40	0.077	-	-	[50]

## Data Availability

The data is available on reasonable request from the corresponding author.
